# Higher remnant cholesterol inflammatory index is associated with increased frailty risk in the UK Biobank

**DOI:** 10.3389/fnut.2026.1864976

**Published:** 2026-07-07

**Authors:** Qianyu Zhou, Mengting Liu, Lianke Wang, Panpan Wang, Ying Qin, Mingyang Zhao, Tong Wanyan, Jinzhou Yu, Yulong Wan, Qiang Zhang, Changqing Sun

**Affiliations:** 1 College of Public Health, Zhengzhou University, Zhengzhou, Henan, China; 2 School of Nursing and Health, Zhengzhou University, Zhengzhou, Henan, China

**Keywords:** frailty, inflammation, lipid metabolism, remnant cholesterol inflammatory index, UK Biobank

## Abstract

**Background:**

Frailty is a major public health concern associated with adverse outcomes. The remnant cholesterol inflammatory index (RCII), a novel biomarker integrating lipid metabolism and systemic inflammation, has been proposed as an indicator of adverse health outcomes. However, little is known about its relationship with frailty. The study aimed to investigate the longitudinal association between RCII and frailty risk in the UK Biobank.

**Methods:**

A total of 402,850 participants from the UK Biobank were included at baseline. RCII was calculated as remnant cholesterol (RC, mg/dL) × C-reactive protein (CRP, mg/L)/10, and frailty was assessed using the Fried frailty phenotype. Cox proportional hazards regression models were applied to evaluate the association between baseline RCII and incident frailty. Restricted cubic spline (RCS) analyses were used to explore potential nonlinear relationships. To capture cumulative exposure, we additionally analyzed 12,895 participants with the same measurements to examine the relationship between cumulative RCII and frailty risk.

**Results:**

During a median follow-up of 15.58 years, 2,327 participants (0.58%) developed frailty. Frailty incidence increased progressively across RCII quartiles, from 0.3% in Q1 to 0.9% in Q4 (*P* for trend <0.001). In the fully adjusted model, each standard deviation increase in RCII was associated with a 11% higher risk of frailty (HR = 1.11, 95% CI: 1.07–1.15). RCS analysis indicated a nonlinear positive relationship, with frailty risk rising more sharply at higher RCII levels. In the 11.68-year subset analysis, 497 participants (3.85%) suffered frailty. Participants in Q4 of cumulative RCII had a significantly higher risk of frailty compared with those in Q1 (HR = 2.34, 95% CI: 1.52–3.60). Subgroup analyses suggested that the association between RCII and frailty was generally consistent across subgroups (*P* for interaction > 0.05).

**Conclusion:**

Higher RCII levels, both at baseline and cumulatively, are associated with an increased risk of frailty. RCII may be a promising biomarker for frailty risk stratification and a potential target for early prevention in aging populations.

## 1 Introduction

Frailty is a common geriatric syndrome characterized by decreased physiological reserve and heightened vulnerability to stressors (1–3), leading to increased risks of disability, hospitalization, and mortality (4, 5). With rapid population aging worldwide, identifying early and modifiable biological determinants of frailty has become a major public health priority.

Remnant cholesterol (RC), representing the cholesterol content of triglyceride-rich lipoproteins (6), has recently emerged as a key contributor to cardiometabolic disorders (7). Elevated RC is strongly associated with systemic inflammation, endothelial injury, and atherosclerosis, even in individuals with normal low-density lipoprotein cholesterol (LDL-C) levels (8–10). Beyond cardiovascular disease, growing evidence suggests that high RC levels are linked to a wide range of adverse health outcomes, including hypertension, stroke, and depressive disorders (11–13). Frailty is strongly influenced by chronic inflammation and metabolic dysregulation. Recent findings further suggest that elevated RC may increase frailty risk among middle-aged and older adults (14). C-reactive protein (CRP), a widely used inflammatory biomarker, is also closely associated with frailty and multiple age-related diseases (15–17). Given that both RC and CRP are individually linked to frailty, it is worthwhile to investigate whether their potential interplay contributes to frailty development and whether integrating these two markers may better capture the underlying metabolic-inflammatory burden.

The remnant cholesterol inflammatory index (RCII) has emerged as a novel biomarker calculated as RC × CRP. Unlike indices based on either lipid or inflammatory markers alone, RCII directly integrates RC, a marker of triglyceride-rich lipoprotein cholesterol burden, with CRP, a widely used marker of systemic inflammation. Therefore, RCII may provide a concise and biologically plausible measure of combined lipid-inflammatory burden, which is highly relevant to frailty development. Recent studies indicate that RCII is associated with adverse cardiometabolic outcomes, including stroke prognosis, diabetic kidney disease, cardiometabolic multimorbidity, and mortality (18–22). However, evidence regarding the role of RCII in frailty remains limited, particularly in large population with long-term follow-up. To address this, the study aimed to investigate the longitudinal association between baseline and long-term cumulative RCII (CumRCII) levels and frailty risk using data from the UK Biobank. Understanding the role of RCII in frailty development may offer new insights into metabolic-inflammatory mechanisms underlying aging and help identify high-risk individuals who may benefit from early preventive strategies.

## 2 Methods

### 2.1 Study population

**Figure 1:**
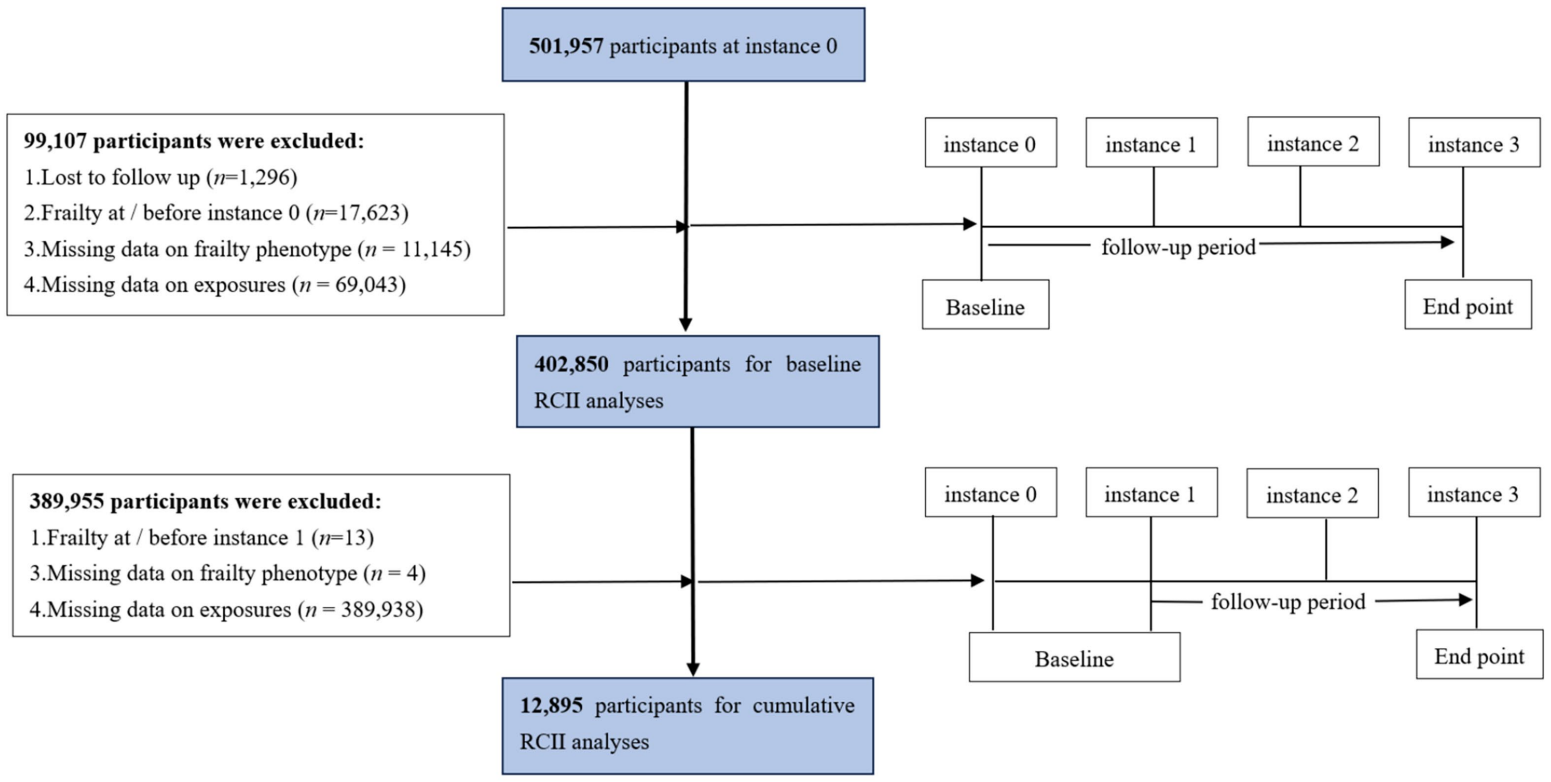
Selection process of the study population.

This longitudinal study used data from the UK Biobank, a large prospective cohort that enrolled 501,957 adults aged 37–73 years between 2006 and 2010 across 22 assessment centers in England, Scotland, and Wales (23). Further details about the UK Biobank have been reported in previous publications (24, 25). Participants completed touchscreen questionnaires to provide sociodemographic, lifestyle, and health information, and underwent physical measurements. In this study, data from instance 0 (baseline, 2006–2010) to instance 3 (the third follow-up, 2019+) were used to evaluate the RCII and CumRCII with frailty risk. For the baseline RCII analysis, participants were followed from instance 0 (baseline) and continued until the first occurrence of frailty, death, loss to follow-up, or the end of follow-up on October 17, 2024, whichever came first. Incident frailty was defined as the first occurrence of frailty identified at any follow-up assessment (instance 1, 2, or 3). For the cumulative RCII analysis, a landmark approach was applied. Participants were followed from instance 1 (the first follow-up) and continued until the first occurrence of frailty, death, loss to follow-up, or the end of follow-up on October 17, 2024, whichever occurred first. Incident frailty was defined as the first occurrence of frailty identified at instance 2 or 3. Details of the study population selection process are provided in Figure 1.

Informed consent was obtained from all participants. The UKB was approved by the North West Multi-Centre Research Ethics Committee (11/NW/0382), and all ethical procedures are overseen by a dedicated Ethics Advisory Committee.^1^ This study was conducted in accordance with the STROBE reporting guidelines (26).

Figure 1 illustrates the selection process of the study population. Of the 501,957 participants in the UK Biobank, we initially excluded 99,107 individuals with missing data on the five frailty components or exposure, lost to follow-up, and those who were frail at baseline, resulting in 402,850 participants included in the baseline RCII analysis. Subsequently, to examine the association between CumRCII and frailty risk, 389,955 participants with missing data on the five frailty components or exposure, as well as those who were frail at instance 1 or earlier, were excluded. Ultimately, 12,895 participants were included in the cumulative RCII analysis. To evaluate potential selection bias, we compared baseline characteristics between participants included in and excluded from the cumulative RCII analysis (see Supplementary Table S1).

### 2.2 Assessment of RCII

Biochemical analyses were conducted at a centralized, accredited laboratory using standardized and quality-controlled methods. Non-fasting venous blood samples were collected at baseline (27). Total cholesterol (TC, data field 30,690) was measured by enzymatic colorimetric assays using a Beckman Coulter AU5800 analyzer. High-density lipoprotein cholesterol (HDL-C, data field 30,760) was determined using an enzyme-immune-inhibition assay, and LDL-C (data field 30,780) was directly measured by an enzymatic selective protection method on the same platform. CRP (data field 30,710) was quantified on the Beckman Coulter AU5800 using a high-sensitivity immunoturbidimetric assay, expressed in mg/L. RC (mg/dL) was calculated as: RC = TC - (HDL-C + LDL-C) (28–30). RCII was calculated as: RCII = RC (mg/dL) × CRP (mg/L)/10 (18, 19). The CumRCII was calculated as: CumRCII = [(RCII _instance 0_ + RCII _instance 1_)/2] × (years attending instance 1 - years attending instance 0), The cumulative RC (CumRC) and CRP (CumCRP) were calculated as: CumRC = [(RC _instance 0_ + RC _instance 1_)/2] × (years attending instance 1 - years attending instance 0), and CumCRP = [(CRP _instance 0_ + CRP _instance 1_)/2] × (years attending instance 1 - years attending instance 0) (18).

### 2.3 Ascertainment of frailty

The outcome of this study was incident frailty, assessed using a modified Fried frailty phenotype adapted for the UK Biobank (31). Compared to other criteria, the frailty phenotype is widely validated in epidemiological research and is cost-effective for large cohort studies (32, 33). It includes five components: weight loss, exhaustion, weakness, walking speed, and physical activity. Weight loss was defined based on self-reported weight change compared with 1 year previously. Exhaustion was assessed using the frequency of feeling tired or having little energy over the past 2 weeks. Weakness was defined using objectively measured grip strength, with sex- and BMI-specific cut-off points. Walking speed was assessed according to self-reported usual walking pace, and physical activity was derived from self-reported participation in different types of physical activity during the previous 4 weeks. Each component was assessed as described in our previous work (25), using detailed structured self-report questionnaires (see Supplementary Table S2). Participants were classified as frail (score ≥3) based on their cumulative scores.

### 2.4 Covariates

Covariates were selected based on prior studies and included sociodemographic, lifestyle, and health-related factors. Demographic variables included sex (female/male), age, ethnicity (White/non-White), Townsend deprivation index (TDI), education level (college/university degree vs. less than college), household income (low, middle, high), and occupational status (working, retired, other). Lifestyle factors comprised physical activity (low, medium, high), smoking status (never, former, current), drinking status (never, former, current), sleep duration (short: 0–6 h, normal: 6–9 h, long: ≥9 h) (34), and body mass index (BMI). Obesity was defined as BMI ≥ 30 kg/m^2^. Health-related factors included diabetes (yes/no), high blood pressure (yes/no), cancer (yes/no), and depression (yes/no), which was assessed using the Patient Health Questionnaire (PHQ)-4 (35). Participants rated four items on a four-point Likert scale from 0 (not at all) to 3 (nearly every day), with a total score of ≥6 indicating emotional disorder. A score ≥3 on items 1 and 2 was considered positive for depression (35).

### 2.5 Statistical analysis

Continuous variables were summarized as mean and standard deviation (SD) or median and interquartile range (IQR), and categorical variables as counts (n) and percentages (%). Group differences were assessed using chi-square tests for categorical variables, one-way ANOVA for normally distributed continuous variables, and Kruskal-Wallis tests for skewed data. RCII, RC, CRP, CumRCII, CumRC, and CumCRP levels were divided into quartiles for group comparisons.

Cumulative frailty risks across RCII and CumRCII levels were estimated using Kaplan–Meier curves, and differences were assessed with the log-rank test. Cox proportional hazards regression was performed to estimate hazard ratios (HRs) and 95% confidence intervals (CIs) using three models: Model 1, unadjusted; Model 2, adjusted for age, sex, occupational status, income, ethnicity, education, TDI, smoking and drinking status, BMI, and physical activity; and Model 3, further adjusted for hypertension, diabetes, cancer, and depression. Restricted cubic spline (RCS) models with four knots were applied based on Model 1 to explore potential nonlinear associations of RCII and CumRCII with incident frailty. Subgroup analyses were conducted based on age, sex, BMI, hypertension, diabetes, and cancer status to examine the impact of baseline RCII and CumRCII on frailty risk and potential interactions. The proportional hazards assumption was confirmed using Schoenfeld residuals. To ensure robust results, several sensitivity analyses were performed: (1) based on the RCS results, RCII and CumRCII were dichotomized for additional Cox regression analyses using the reference points corresponding to HR = 1, which were identified at RCII = 3.32 and CumRCII = 13.60, respectively; (2) missing data were imputed using chained equations for multiple imputations; (3) considering the competing risk between mortality and incident frailty, we repeated the main analyses using the Fine-Gray competing risk models, with death treated as a competing event. All statistical analyses were performed in R version 4.2.3, and two-sided *p* < 0.05 were considered statistically significant.

## 3 Results

### 3.1 Baseline characteristics

A total of 402,850 participants from the UK Biobank were included in the final analyses. Baseline characteristics of the study population according to RCII quartiles are shown in Table 1. The mean age of the participants was (56.50 ± 8.10) years, and 215,579 (53.50%) were female. During a median follow-up of 15.58 years, 2,327 participants (0.58%) developed frailty. Participants with higher RCII levels tended to be older, more likely to be male, have lower educational attainment and household income, engage in less physical activity, and have higher rates of smoking and obesity (all *p* < 0.001). They also exhibited lower HDL-C and higher LDL-C, total cholesterol, CRP, RC, and RCII levels (all *p* < 0.001). In addition, the proportion of hypertension, diabetes, cancer, and depression increased progressively across RCII quartiles (all *p* < 0.001).

**Table 1:** Baseline characteristics of participants according to the quartile of RCII.

Characteristics	Total (*N* = 402,850)	Q1: ≤ 1.41 (*n* = 100,714)	Q2: 1.41–3.26 (*n* = 100,711)	Q3: 3.26–7.47 (*n* = 100,714)	Q4: > 7.47 (*n* = 100,711)	*P*
Age, mean (SD), years	56.50 (8.10)	54.70 (8.31)	56.60 (8.04)	57.20 (7.91)	54.70 (7.84)	<0.001
Female, *n* (%)	215,579 (53.50)	58,181 (57.77)	51,429 (51.07)	50,694 (50.33)	55,275 (54.88)	<0.001
White, *n* (%)	382.235 (94.90)	94,997 (94.32)	95,655 (94.98)	95,790 (95.11)	95,793 (95.12)	<0.001
College/University degree, *n* (%)	133,255 (33.10)	41,955 (41.66)	34,653 (34.41)	30,774 (30.56)	25,873 (25.69)	<0.001
Household income, *n* (%)						<0.001
Low (<£51,999)	255,075 (63.30)	59,144 (58.72)	63,113 (62.67)	65,094 (64.63)	67,724 (67.25)	
Middle (£52,000–£100,000)	72,714 (18.00)	22,143 (21.99)	18,982 (18.85)	17,276 (17.15)	14,313 (14.21)	
High (>£100,000)	19,373 (4.81)	6,922 (6.87)	5,168 (5.13)	4,126 (4.10)	3,157 (3.13)	
Occupational status, *n* (%)						<0.001
Working	247,033 (61.30)	69,000 (68.51)	62,491 (62.05)	59,570 (59.15)	55,972 (55.58)	
Retired	126,777 (31.50)	24,886 (24.71)	31,858 (31.63)	34,236 (33.99)	35,797 (35.54)	
Other	25,536 (6.34)	5,924 (5.88)	5,515 (5.48)	6,068 (6.02)	8,029 (7.97)	
TDI, median (IQR)	−2.23 (4.02)	- 2.32 (3.88)	−2.35 (3.83)	−2.25 (3.97)	−1.97 (4.38)	<0.001
Sleep duration, h, *n* (%)						<0.001
Short: 0 ~ 6	20,016 (4.97)	4,364 (4.33)	4,679 (4.65)	4,994 (4.96)	5,979 (5.94)	
Normal: 6 ~ 9	351,953 (87.40)	90,248 (89.61)	88,933 (88.31)	87,725 (87.10)	85,047 (84.45)	
Long: ≥9	28,970 (7.19)	5,753 (5.71)	6,658 (6.61)	7,490 (7.44)	9,069 (9.00)	
Smoking status, *n* (%)						<0.001
Never	221,017 (54.90)	60,633 (60.20)	56,880 (56.48)	54,055 (53.67)	49,449 (49.10)	
Former	139,721 (34.70)	32,357 (32.13)	34,651 (34.41)	36,049 (35.79)	36,664 (36.41)	
Current	40,783 (10.10)	7,465 (7.41)	8,842 (8.78)	10,283 (10.21)	14,193 (14.09)	
Drinking status, *n* (%)						<0.001
Never	16,088 (3.99)	3,472 (3.45)	3,690 (3.66)	4,057 (4.03)	4,869 (4.83)	
Former	13,071 (3.24)	2,938 (2.92)	2,923 (2.90)	3,278 (3.25)	3,932 (3.90)	
Current	373,345 (92.70)	94,233 (93.56)	94,016 (93.35)	93,296 (92.63)	91,800 (91.15)	
Physical activities, *n* (%)						<0.001
Low	54,437 (13.50)	11,034 (10.96)	12,575 (12.49)	14,313 (14.21)	16,515 (16.40)	
Moderate	129,361 (32.10)	32,866 (32.63)	32,832 (32.60)	32,309 (32.08)	31,354 (31.13)	
High	131,891 (32.70)	38,218 (37.95)	34,668 (34.42)	31,802 (31.58)	27,203 (27.01)	
BMI ≥ 30 kg/m^2^, *n* (%)	93,715 (23.30)	6,204 (6.16)	15,836 (15.72)	27,574 (27.38)	44,101 (43.79)	<0.001
Diabetes, *n* (%)	18,553 (4.61)	4,250 (4.22)	4,484 (4.45)	4,620 (4.59)	5,199 (5.16)	<0.001
High blood pressure, *n* (%)	162,234 (40.30)	30,359 (30.14)	39,456 (39.18)	44,350 (44.04)	48,069 (47.73)	<0.001
Depression, *n* (%)	18,928 (4.70)	4,327 (4.30)	4,215 (4.19)	4,648 (4.62)	5,738 (5.70)	<0.001
Cancer, *n* (%)	29,898 (7.42)	6,486 (6.44)	7,131 (7.08)	7,609 (7.56)	8,672 (8.61)	<0.001
HDL-C, mean (SD), mmol/L	1.45 (0.38)	1.61 (0.40)	1.48 (0.38)	1.40 (0.35)	1.33 (0.33)	<0.001
LDL-C, mean (SD), mmol/L	3.57 (0.86)	3.20 (0.75)	3.52 (0.81)	3.70 (0.85)	3.87 (0.89)	<0.001
Cholesterol, mean (SD), mmol/L	5.71 (1.14)	5.29 (1.01)	5.64 (1.07)	5.85 (1.11)	6.06 (1.19)	<0.001
CRP, median (IQR), mg/L	1.30 (2.02)	0.41 (0.29)	0.92 (0.50)	1.77 (0.96)	4.43 (4.10)	<0.001
RC, median (IQR), mg/dL	24.90 (13.69)	18.21 (9.59)	23.82 (10.98)	27.57 (12.49)	31.63 (15.12)	<0.001
RCII, median (IQR)	3.26 (6.05)	0.80 (0.58)	2.19 (0.91)	4.83 (1.99)	13.34 (11.82)	<0.001

TDI, Townsend deprivation index; BMI, body mass index; SBP, systolic blood pressure; HDL-C, high-density lipoprotein cholesterol; LDL-C, low-density lipoprotein direct; Medication, medication for cholesterol, blood pressure or diabetes; CRP, C-reactive protein; RC, remnant cholesterol; RCII, remnant cholesterol inflammatory index.

### 3.2 Association between RCII and incident frailty

**Figure 2:**
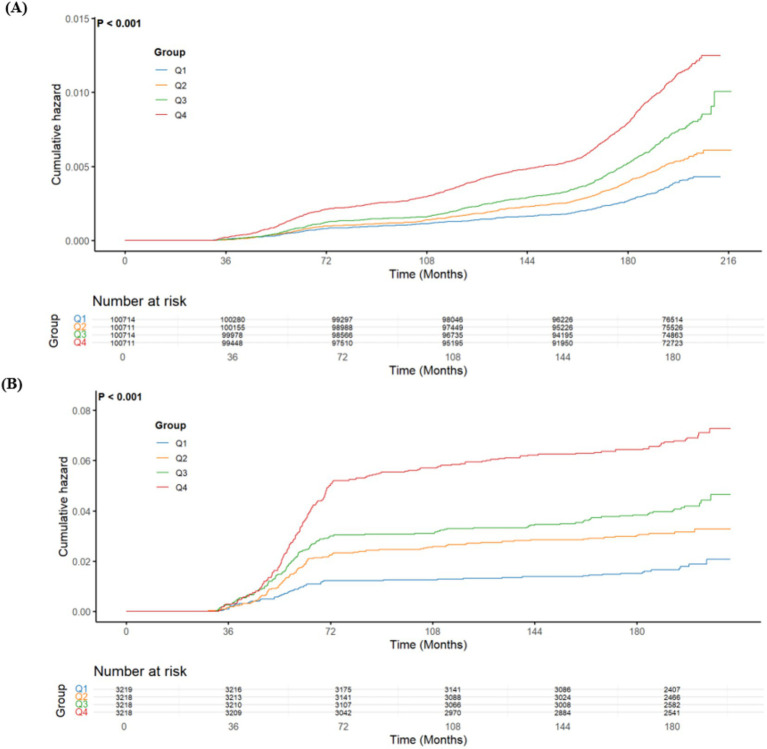
K-M plot of cumulative incidence of new-onset frailty based on RCII and CumRCII levels. **(A)** Based on RCII levels. **(B)** Based on CumRCII levels.

Figure 2A shows the Kaplan–Meier curves for frailty across RCII quartiles. The estimated cumulative incidence of frailty increased progressively from 0.3% in Q1 to 0.5% in Q2, 0.6% in Q3, and 0.9% in Q4. The log-rank test indicated significant differences in frailty incidence among quartiles (*p* < 0.001), demonstrating a graded increase in frailty risk with higher RCII levels.

**Figure 3:**
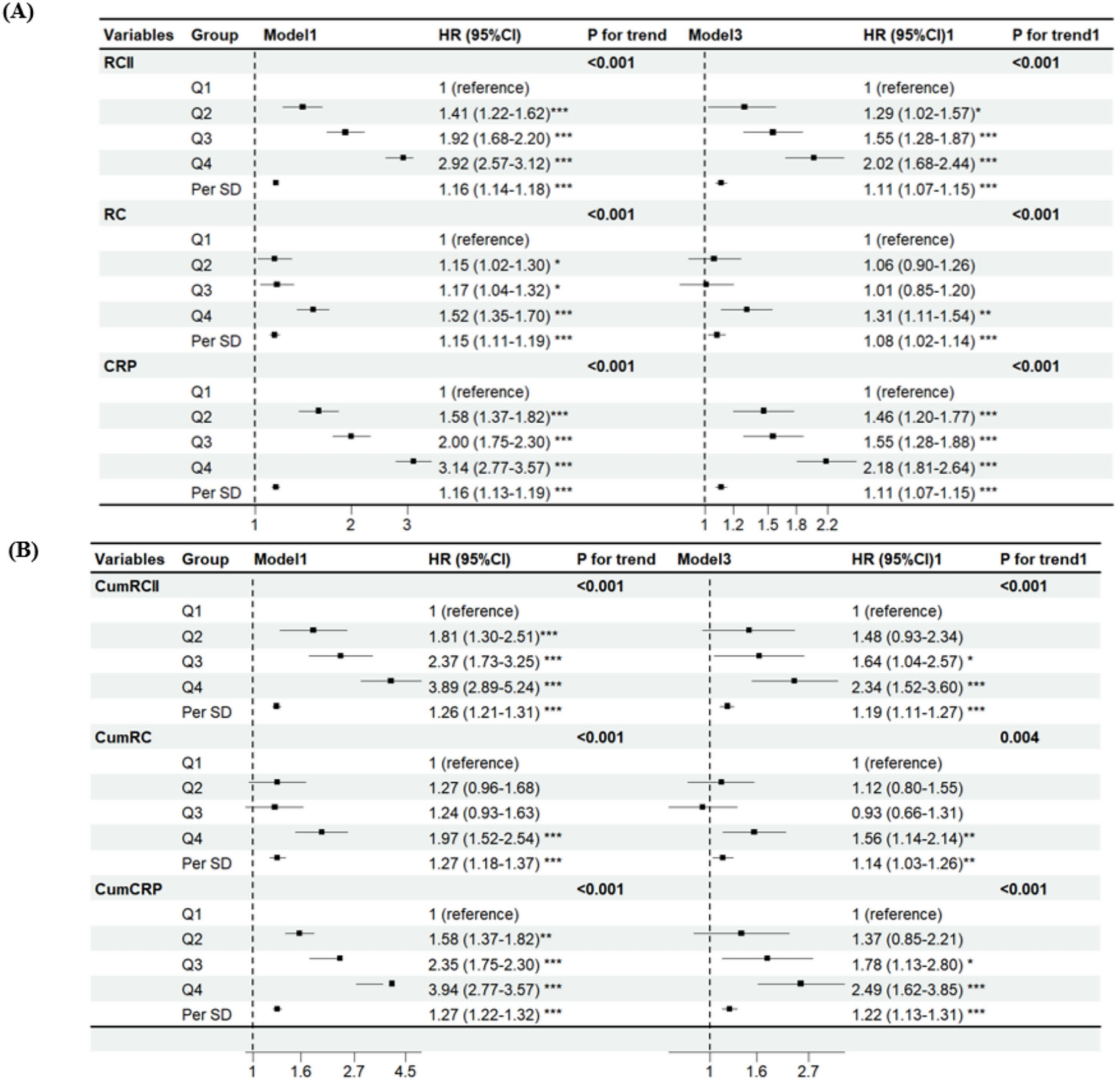
Associations of exposures with the risk of frailty. **(A)** Associations of RC, CRP, and RCII with the risk of frailty. **(B)** Associations of CumRC, CumCRP, and CumRCII with the risk of frailty. Model 1 unadjusted; Model 3 adjusted for age, sex, occupational status, income, ethnics, education, TDI, drinking status, smoking status, BMI, physical activity, high blood pressure, diabetes, cancer and depression. ***means *P* < 0.001, **means *P* < 0.01, and *means *P* < 0.05.

The association between RCII and frailty risk is shown in Figure 3A. Each SD increase in RCII was associated with a higher risk of frailty, with an unadjusted HR of 1.16 (95% CI, 1.14–1.18) and a fully adjusted HR of 1.11 (95% CI, 1.07–1.15). Similarly, per SD increase in RC and CRP was associated with 8% (HR = 1.08, 95% CI: 1.02–1.14) and 11% (HR = 1.11, 95% CI: 1.07–1.15) higher frailty risk, respectively, in fully adjusted models.

As expected, when RCII Q1 was used as the reference, participants in Q2, Q3 and Q4 exhibited significantly higher frailty risks in the adjusted models (Q2: HR = 1.29, 95% CI: 1.02–1.57; Q3: HR = 1.55, 95% CI: 1.28–1.87; Q4: HR = 2.02, 95% CI: 1.68–2.44). Stratification by CRP quartiles revealed similar findings. Compared with Q1, higher frailty risks were observed in Q2 (HR = 1.46, 95% CI: 1.20–1.77), Q3 (HR = 1.55, 95% CI: 1.28–1.88), and Q4 (HR = 2.18, 95% CI: 1.81–2.64). In contrast, only the highest RC quartile (Q4) showed a significant association with frailty in the fully adjusted models (HR = 1.31, 95% CI: 1.11–1.54), while significant trends were observed in unadjusted analyses.

### 3.3 Association between CumRCII and incident frailty

Figure 2B shows the Kaplan–Meier curves for frailty across CumRCII quartiles. The estimated cumulative incidence of frailty increased progressively from 1.7% in Q1 to 3.1% in Q2, 4.0% in Q3, and 6.6% in Q4. The log-rank test indicated significant differences in frailty incidence among quartiles (*p* < 0.001). The subset analysis involved 12,895 participants with a mean age of 57.26 ± 7.38 years, among whom 497 participants (3.85%) developed frailty during a median follow-up of 11.68 years (Supplementary Table S3). Compared with participants excluded from the cumulative RCII analysis, those included in this analysis showed some differences in baseline characteristics, particularly in socioeconomic status, ethnicity, smoking status, employment status, age, and sex, while most clinical and biochemical variables showed relatively small standardized differences (see Supplementary Table S1).

Figure 3B presents the associations between CumRCII, CumRC, and CumCRP and frailty incidence. Per SD increase in CumRCII was associated with 19% higher risk of frailty in the fully adjusted model (HR = 1.19, 95% CI: 1.11–1.27). Similarly, per SD increase in CumRC and CumCRP was associated with 14% (HR = 1.14, 95% CI: 1.03–1.26) and 22% (HR = 1.22, 95% CI: 1.13–1.31) higher frailty risk, respectively, in fully adjusted models.

As expected, when using CumRCII Q1 as the reference, participants in Q3 and Q4 had significantly higher frailty risks in adjusted models (Q3: HR = 1.64, 95% CI: 1.04–2.57; Q4: HR = 2.34, 95% CI: 1.52–3.60), whereas Q2 showed no significant association. Stratification by CumCRP quartiles revealed similar findings. Compared with Q1, Q3 (HR = 1.78, 95% CI: 1.13–2.80) and Q4 (HR = 2.49, 95% CI: 1.62–3.85) were associated with higher frailty risk, while Q2 remained non-significant. For CumRC, only the highest quartile (Q4) showed a significant association with frailty, both in unadjusted (HR = 1.97, 95% CI: 1.52–2.54) and fully adjusted models (HR = 1.56, 95% CI: 1.14–2.14).

### 3.4 Non-linear relationship of RCII and CumRCII with frailty

**Figure 4:**
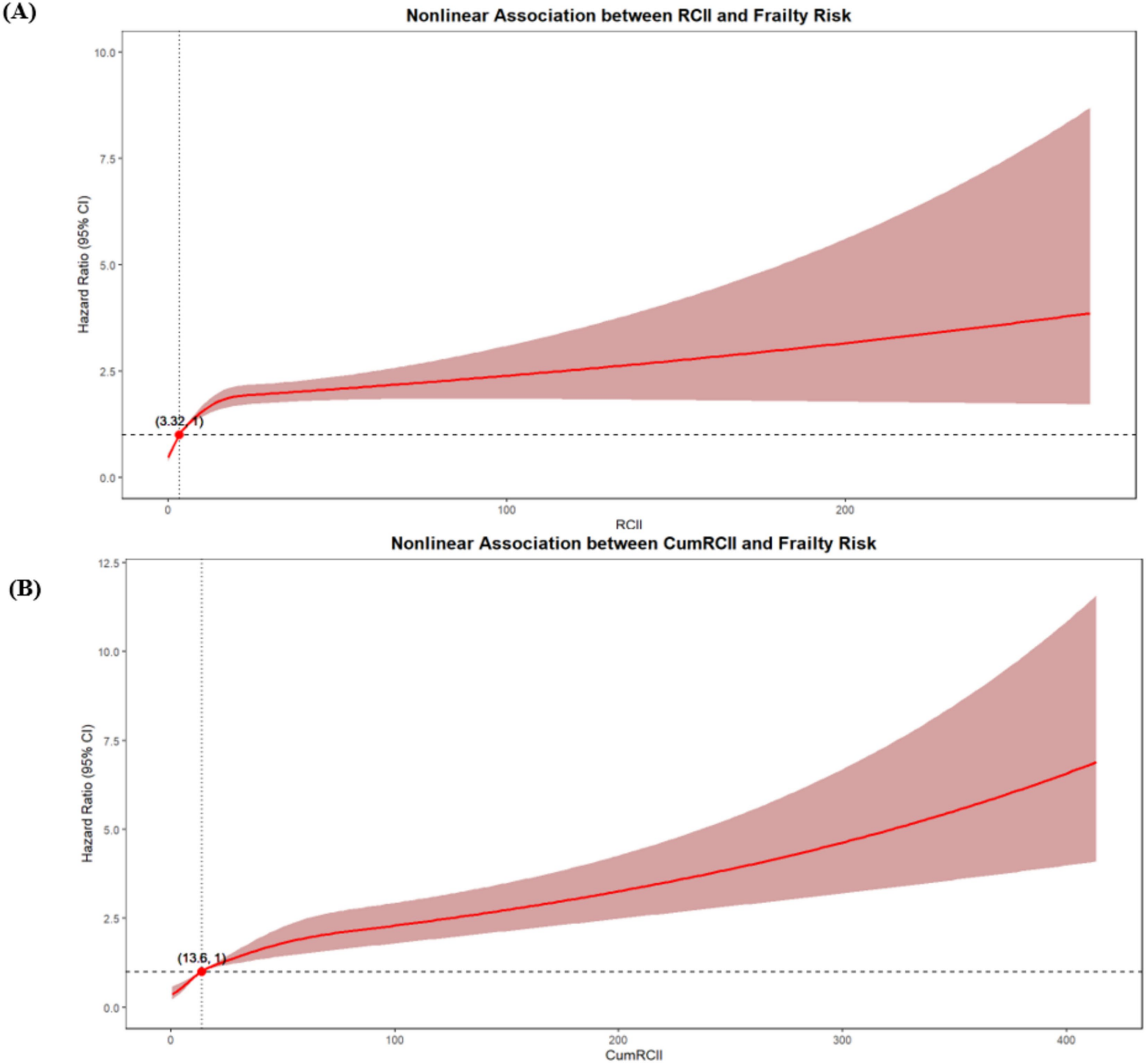
Associations between RCII and CumRCII and frailty risk using restricted cubic splines. **(A)** Associations between RCII and frailty risk. **(B)** Associations between CumRCII and frailty risk. The figure displays the unadjusted hazard ratio (HR, solid lines) with 95% confidence intervals (CI, shaded areas).

Figures 4A,B illustrate the association of RCII and CumRCII with frailty risk using RCS. A significant non-linear relationship was observed (*P* for nonlinearity < 0.001), with frailty risk increasing more sharply at higher RCII and CumRCII levels. The reference point (HR = 1) was set at RCII = 3.32 and CumRCII = 13.6.

### 3.5 Subgroup and sensitivity analysis

**Figure 5:**
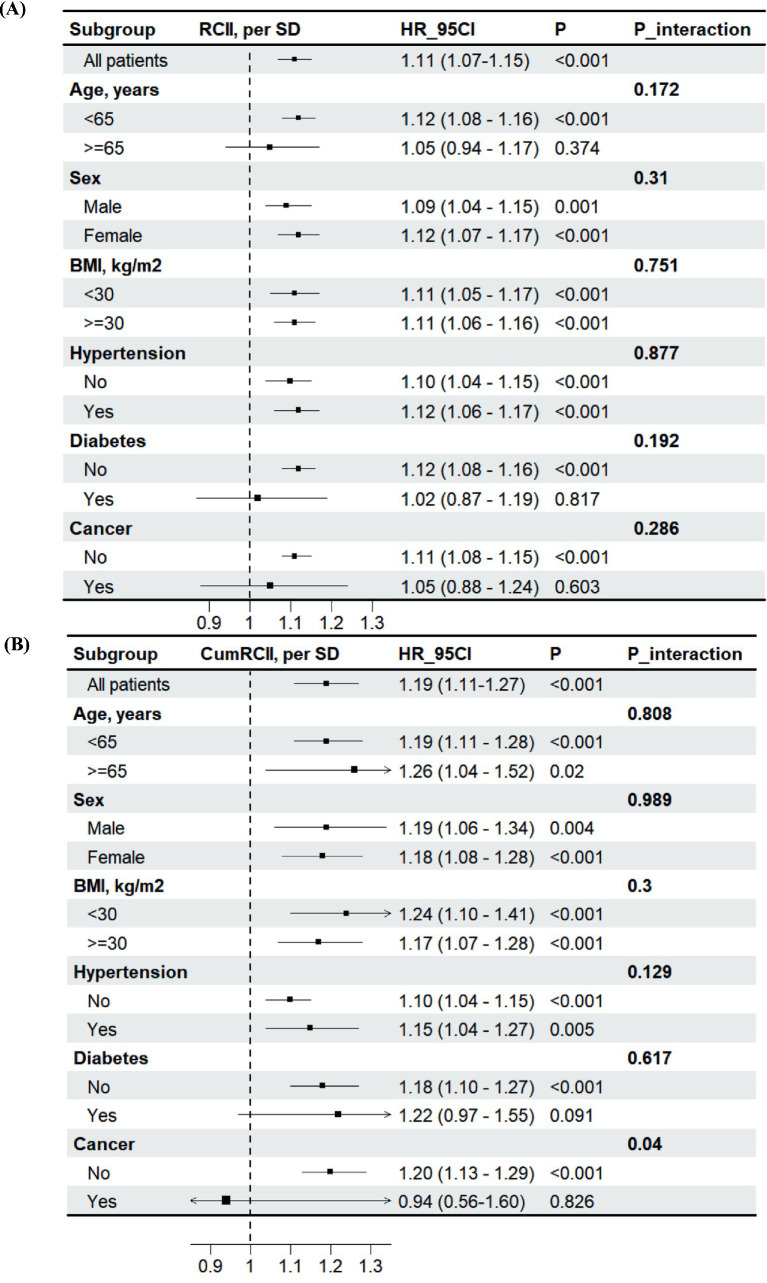
Subgroup analysis of the relationship between RCII and CumRCII and frailty. **(A)** Subgroup analysis between RCII and frailty. **(B)** Subgroup analysis between CumRCII and frailty. HRs were calculated to evaluate the association between each SD increase in RCII and frailty, with adjustments for covariates included in Model 3.

Figures 5A,B show the associations of RCII and CumRCII with frailty risk, stratified by age, sex, BMI, hypertension, diabetes, and cancer status, respectively. HRs and 95%CIs were estimated per SD increase in RCII and CumRCII using fully adjusted models. For RCII, no significant interactions were observed across subgroups (all *P* for interaction > 0.05), although the association was not statistically significant in some subgroups, including participants aged ≥ 65 years or those with diabetes or cancer. For CumRCII, the positive association with frailty risk remained significant across most subgroups. A significant interaction was detected for cancer status (*P* for interaction = 0.04), with a stronger association observed among participants without cancer, whereas no significant association was found among those with cancer.

Sensitivity analyses yielded results consistent with the main analyses after imputing missing data (Supplementary Tables S4, S5) and conducting the competing risk analyses (Supplementary Tables S6, S7). Additionally, when analyzing the association of RCII and CumRCII with frailty risk using the data-driven thresholds derived from the RCS analysis, participants with RCII ≥ 3.32 had a 5% higher risk of frailty compared with those with RCII < 3.32 (HR = 1.05, 95% CI: 1.02–1.08), while participants with CumRCII ≥ 13.60 had a 61% higher risk compared with those with CumRCII < 13.60 (HR = 1.61, 95% CI: 1.22–2.12) (Supplementary Table S8).

## 4 Discussion

In this large prospective study based on the UK Biobank, we found that higher RCII levels were significantly associated with an increased risk of incident frailty. Both baseline and CumRCII were positively associated with frailty development, with risks increasing by 2.02-fold and 2.34-fold, respectively. However, the association per SD increase in baseline RCII was relatively modest (HR = 1.11), indicating that statistical significance does not necessarily imply strong clinical predictive value. RCS analyses demonstrated a nonlinear dose–response relationship, indicating that frailty risk increased more steeply at higher RCII levels. Subgroup analyses further demonstrated that the association between RCII and frailty was generally consistent across major subgroups, with limited evidence of effect modification, except for cancer status in the CumRCII analysis. In addition, multiple sensitivity analyses confirmed the robustness of our findings. To our knowledge, this study provides the first comprehensive evaluation of both baseline and cumulative RCII in relation to frailty risk within a large population-based cohort. These findings suggest that RCII may help characterize metabolic-inflammatory pathways related to frailty, although its standalone utility for individual-level risk stratification requires further validation.

Traditionally, frailty has been associated with chronic inflammation, metabolic dysregulation, and impaired energy homeostasis, yet the underlying biological mechanisms remain incompletely understood (36, 37). Frailty is a multidimensional geriatric syndrome driven by these interrelated processes. Both lipid abnormalities and low-grade inflammation have been implicated in its pathogenesis. RC, reflecting the cholesterol content in triglyceride-rich lipoproteins, contributes to endothelial injury, oxidative stress, and systemic inflammation. Meanwhile, CRP is a sensitive biomarker of inflammatory activation and is closely linked to functional decline. Previous research has shown that combining RC with CRP improves risk stratification (38–40). This concept underlies the development of the RCII, an integrated index that incorporates both metabolic and inflammatory risk markers to enhance predictive capacity (18). Earlier studies on RCII primarily focused on stroke risk, short-term prognosis in acute ischemic stroke patients, dementia, and all-cause or cause-specific mortality (18–20, 41). Building on this evidence, we introduced RCII as a comprehensive index capturing the interaction between metabolic and inflammatory pathways in frailty development. The observed association supports the potential relevance of evaluating these pathways jointly.

The mechanisms linking RCII to frailty are likely multifactorial. Elevated RC may contribute to the development of frailty by impairing skeletal muscle metabolism and promoting systemic inflammation. Lipid metabolites from remnant lipoproteins disrupt insulin signaling, activate FoxO3, and induce muscle protein breakdown, leading to mitochondrial dysfunction and reduced muscle mass (42–44). Concurrently, RC-associated low-grade inflammation stimulates pro-inflammatory cytokines that suppress IGF-1 signaling and accelerate sarcopenia (45, 46). Elevated CRP reflects this chronic inflammatory state, further exacerbating hormonal dysregulation, appetite loss, and functional decline. Together, these metabolic and inflammatory insults diminish physical resilience and increase vulnerability to stressors, key features of frailty. The non-linear relationship observed in our RCS analysis may reflect threshold effects, whereby metabolic-inflammatory burden exerts minimal influence below a certain level but triggers accelerated physiological decline once cumulative exposure becomes substantial. This underscores the importance of early identification and management of individuals with elevated RCII levels before frailty accelerates.

Subgroup analyses showed that the association between RCII and frailty was generally consistent across most participant characteristics, suggesting that the relationship between metabolic-inflammatory burden and frailty risk is relatively stable across different demographic and clinical backgrounds. Interestingly, a potential effect modification by cancer status was observed in the CumRCII analysis. This finding may reflect the complex metabolic and inflammatory alterations associated with cancer and its treatments. Cancer and anticancer therapies are known to induce systemic inflammation, metabolic disturbances, and accelerated muscle wasting, which may interact with lipid-inflammatory pathways and amplify physiological vulnerability (47, 48). Consequently, individuals with cancer may experience a different trajectory of metabolic-inflammatory exposure and frailty progression compared with those without cancer. In addition, competing health risks and treatment-related factors in cancer patients may further influence the observed associations (49). However, given the exploratory nature of subgroup analyses, these findings should be interpreted with caution and warrant further investigation in future studies.

The CumRCII analysis further highlights the importance of long-term metabolic and inflammatory exposure in the development of frailty. While baseline RCII reflects the metabolic-inflammatory status at a single time point, CumRCII may better capture the sustained physiological burden resulting from prolonged exposure to lipid abnormalities and chronic inflammation. Frailty is widely recognized as a progressive condition characterized by the gradual accumulation of physiological deficits over time (50). Therefore, long-term exposure to adverse metabolic and inflammatory states may play a critical role in accelerating this process. Previous studies have demonstrated the value of cumulative RC exposure in predicting incident hypertension (11). Extending these findings, our study suggests that persistent metabolic-inflammatory burden, as reflected by cumulative RCII, may also contribute to frailty development. These results underscore the potential value of monitoring RCII trajectories over time rather than relying solely on a single measurement.

Our findings have potential clinical and public health implications. RCII can be readily derived from routinely measured lipid profiles and CRP levels, making it a practical marker for reflecting combined lipid-inflammatory burden. However, given the modest effect size observed for baseline RCII per SD increase, RCII should not be regarded as a standalone clinical prediction tool for frailty at this stage. Instead, it may provide additional information on metabolic-inflammatory risk when considered together with established frailty risk factors. Future studies are warranted to validate these findings in diverse populations and to evaluate whether RCII improves frailty risk prediction beyond conventional demographic, lifestyle, and clinical factors.

Nevertheless, several potential limitations of our study should be acknowledged. First, as noted previously (51), the UK Biobank population is known to be healthier and more socioeconomically advantaged than the general population, which may limit the generalizability of our findings. In addition, the mean age at baseline was approximately 56 years, which is relatively young for frailty research. Therefore, the results may be more applicable to middle-aged and early older adults and should be generalized cautiously to older, frailer, or more socioeconomically disadvantaged populations. Second, residual confounding cannot be fully ruled out, despite extensive adjustments for sociodemographic, lifestyle, and health-related factors. In particular, dietary patterns, medication use such as statins or other lipid-lowering therapies, multimorbidity burden, and other inflammatory conditions were not fully incorporated into the primary models. These factors may influence both lipid-inflammatory status and frailty risk and could have affected the observed associations. Moreover, lipid-lowering medications may directly affect RCII levels; therefore, further studies are needed to examine whether medication use modifies or attenuates the association between RCII and frailty. Third, frailty was assessed using a modified Fried frailty phenotype adapted for the UK Biobank. Although the weakness component was based on objectively measured grip strength, several other components, including weight loss, exhaustion, walking speed, and physical activity, were derived from structured self-reported questionnaire data. Therefore, this modified phenotype differs from the original Fried phenotype and may be subject to recall and reporting biases. Such bias may have introduced non-differential misclassification of frailty status, potentially attenuating the observed associations toward the null, which is a common limitation in large-scale epidemiological studies. Fourth, the cumulative RCII analysis included only participants with repeated RCII measurements at instance 0 and instance 1 and available subsequent frailty assessment data. This resulted in a substantially smaller analytic sample and may have introduced selection bias, as participants with repeated measurements differed from those without such data in several baseline characteristics. Therefore, the findings from the cumulative RCII analysis should be interpreted with caution and require validation in other cohorts with repeated biomarker measurements. Finally, mechanistic pathways could not be directly assessed, underscoring the need for future experimental studies to elucidate the biological links between RCII and frailty.

## 5 Conclusion

In summary, this study demonstrates that higher baseline and cumulative RCII levels are significantly associated with increased frailty risk. RCII may help characterize metabolic-inflammatory risk related to frailty, although its incremental predictive value and clinical applicability require further validation.

## Data Availability

The data used in this study were obtained from the UK Biobank Resource (https://www.ukbiobank.ac.uk/), approved application number 772449. Researchers may apply to access the UK Biobank data through the official UK Biobank application process.

## Footnotes

1
http://www.ukbiobank.ac.uk/ethics
