# Supplementary material for: Higher remnant cholesterol inflammatory index is associated with increased frailty risk in the UK Biobank
Source: Front Nutr. 2026 Jul 7;13:1864976. doi: 10.3389/fnut.2026.1864976 (PMC13385681; doi:10.3389/fnut.2026.1864976)
Supplement: Supplementary file 1 [file Data_Sheet_1.pdf]

Supplementary information

Table S1. Comparison of baseline characteristics between participants included in and excluded from the cumulative RCII analysis

Table S2. The 5 criteria for the frailty phenotype in the UK Biobank

Table S3. Baseline characteristics of participants according to the quartile of CumRCII

Table S4. Associations of RC, CRP, and RCII with the risk of frailty after being imputed

Table S5. Associations of CumRC, CumCRP, and CumRCII with the risk of frailty after being imputed

Table S6. Association of RC, CRP, and RCII with frailty risks when using the competing risk model

Table S7. Association of CumRC, CumCRP, and CumRCII with frailty risks when using the competing risk model

Table S8. Association of RCII and CumRCII with frailty risk based on restricted cubic splines categories

Table S1 Baseline characteristics of participants according to the quartile of CumRCII.

**Characteristics**	**Included (n = 12,895)**	**Excluded due to missing frailty data (*n* = 389,955)**	***P***	***SMD***
Age, mean (SD), years	57.30 (7.38)	56.50 (8.12)	<0.001	0.101
Female, *n* (%)	6,241 (48.4)	209,338 (53.70)	<0.001	0.106
White, *n* (%)	12,600 (97.70)	369,635 (94.80)	<0.001	0.153
College/University degree, *n* (%)	5,564 (43.20)	127,691 (32.80)	<0.001	0.162
Household income, *n* (%)			<0.001	0.043
Low (<£51,999)	8,535 (66.20)	246,540 (63.20)		
Middle (£52,000-£100,000)	2,572 (20.00)	70,142 (18.00)		
High (>£100,000)	562 (4.36)	18,811 (4.82)		
Occupational status, *n* (%)			<0.001	0.113
Working	7,978 (61.90)	239,055 (61.30)		
Retired	4,318 (33.50)	122,459 (31.40)		
Other	516 (4.00)	25,020 (6.42)		
TDI, median (IQR)	-2.76 (3.15)	- 2.21 (4.05)	<0.001	0.243
Sleep duration, h, *n* (%)			<0.001	0.069
Short: 0~6	473 (3.67)	19,543 (5.01)		
Normal: 6~9	11,499 (89.20)	340,454 (87.30)		
Long: ≥9	879 (6.82)	28,091 (7.20)		
Smoking status, *n* (%)			<0.001	0.151
Never	7,632 (59.20)	213,385 (54.70)		
Former	4,422 (34.30)	135,299 (34.70)		
Current	809 (6.27)	39974 (10.20)		
Drinking status, *n* (%)			<0.001	0.091
Never	356 (2.76)	15,732 (4.03)		
Former	305 (2.37)	12,766 (3.27)		
Current	12,230 (94.80)	361,115 (92.60)		
Physical activities, *n* (%)			<0.001	0.050
Low	1,983 (15.40)	52,454 (13.40)		
Moderate	4,436 (34.40)	124,925 (32.00)		
High	4,218 (32.70)	127,673 (32.70)		
BMI ≥ 30kg/m^2, *n* (%)	2,549 (19.80)	91,166 (23.40)	<0.001	0.088
Diabetes, *n* (%)	482 (3.74)	18,071 (4.63)	<0.001	0.045

High blood pressure, *n* (%)	5,405 (41.90)	156,829 (40.20)	0.682	0.004
Depression, *n* (%)	407 (3.16)	18,521 (4.75)	<0.001	0.088
Cancer, *n* (%)	823 (6.38)	29,075 (7.46)	<0.001	0.042
HDL-C, mean (SD), mmol/L	1.45 (0.37)	1.45 (0.38)	0.275	0.010
LDL-C, mean (SD), mmol/L	3.55 (0.85)	3.57 (0.86)	0.002	0.028
Cholesterol, mean (SD), mmol/L	5.67 (1.13)	5.71 (1.14)	<0.001	0.035
CRP, median (IQR), mg/L	1.16 (1.75)	1.3 (2.03)	<0.001	0.058
RC, median (IQR), mg/dL	24.60 (13.40)	24.90 (13.70)	<0.001	0.041
RCII, median (IQR)	2.91 (5.18)	3.27 (6.09)	<0.001	0.071

TDI, Townsend deprivation index; BMI, body mass index; SBP, systolic blood pressure; HDL-C, high-density lipoprotein cholesterol; LDL-C, low-density lipoprotein direct; Medication, medication for cholesterol, blood pressure or diabetes; CRP, C-reactive protein; RC, remnant cholesterol; RCII, remnant cholesterol inflammatory index; SMD, standardized mean difference.

Table S2. The 5 criteria for the frailty phenotype in the UK Biobank.

Indicators	Criteria	Field ID
Weight loss	Question: Compared with one year ago, has your weight changed?<br>• Yes, lost weight=1;<br>• Others=0; Do not know=0;<br>• Prefer not to answer=excluded	2306
Exhaustion	Question: Over the past 2 weeks, how often have you felt tired or had little energy?<br>• More than half the days or nearly every day =1;<br>• Others=0; Do not know=0<br>• Prefer not to answer=excluded	2080
Weakness	Measured grip strength expressed in kg by sex- and BMI-adjusted cut-off points:<br>• Males:<br>BMI≤24 & grip strength≤29;<br>24.1≤BMI≤28 & grip strength≤30;<br>BMI>28 & grip strength≤32<br>• Females:<br>BMI≤23 & grip strength≤17;<br>23.1≤BMI≤26 & grip strength≤17.3;<br>26.1≤BMI≤29 & grip strength≤18;<br>BMI>29 & grip strength≤21	46<br>47
Walking speed	Question: How would you describe your usual walking pace?"<br>• Slow pace=1;<br>• Others=0;<br>• None of the above/Prefer not to answer=excluded	924
Physical activity	Question: In the last 4 weeks, did you spend any time doing the following?<br>• Walking for pleasure =0;<br>• Strenuous sports=0;<br>• Light DIY (eg, pruning, watering the lawn):<br>Frequency of once per week or less=1;<br>Frequency of more than once per week=0;<br>• Heavy DIY (eg, weeding, lawn mowing, carpentry, digging) =0;<br>• Other exercises (eg: swimming, cycling, keep fit, bowling) =0;<br>• None of the above=1;<br>• Prefer not to answer=excluded	6164<br>1011

Table S3. Baseline characteristics of participants according to the quartile of CumRCII.

**Characteristics**	**Total** (***N*** **= 12,895)**	**Q1: ≤ 6.23** **(*n* =3,219)**	**Q2: 6.23-13.56** **(*n* =3,218)**	**Q3: 13.56-28.90** **(*n* =3,218)**	**Q4: > 28.90** **(*n* =3,218)**	***P***
Age, mean (SD), years	57.26 (7.38)	56.10 (7.78)	57.50 (7.32)	57.90 (7.01)	57.60 (7.24)	<0.001
Female, *n* (%)	6,241 (48.40)	1,656 (51.44)	1,497 (46.52)	1,469 (45.65)	1,607 (49.94)	<0.001
White, *n* (%)	12,600 (97.70)	3,148 (97.79)	3,134 (97.39)	3,141 (97.61)	3,156 (98.07)	0.132
College/University degree, *n* (%)	5,564 (43.10)	1,630 (50.64)	1,482 (46.05)	1,307 (40.62)	1,138 (35.36)	<0.001
Household income, *n* (%)						<0.001
Low (<£51,999)	8,535 (66.20)	2,026 (62.94)	2,104 (65.38)	2,134 (66.31)	2,257 (70.14)	
Middle (£52,000–£100,000)	2,572 (19.90)	716 (22.24)	674 (20.94)	649 (20.17)	530 (16.47)	
High (>£100,000)	562 (4.36)	191 (5.93)	150 (4.66)	116 (3.60)	104 (3.23)	
Occupational status, *n* (%)						<0.001
Working	7,978 (61.90)	2,110 (65.55)	1,976 (61.40)	1,931 (60.01)	1,949 (60.57)	
Retired	4,318 (33.50)	953 (29.61)	1,099 (34.15)	1,156 (35.92)	1,101 (34.21)	
Other	516 (4.00)	133 (4.13)	122 (3.79)	111 (3.45)	149 (4.63)	
TDI, median (IQR)	-2.76 (3.15)	- 2.90 (3.01)	-2.79 (2.95)	-2.81 (3.10)	-2.56 (3.48)	<0.001
Sleep duration, h, *n* (%)						<0.001
Short: 0~6	473 (3.67)	105 (3.26)	88 (2.73)	132 (4.10)	148 (4.60)	
Normal: 6~9	11,499 (89.20)	2,912 (90.46)	2,914 (90.55)	2,870 (89.19)	2,782 (86.45)	
Long: ≥9	879 (6.82)	190 (5.90)	203 (6.31)	206 (6.40)	279 (8.67)	
Smoking status, *n* (%)						<0.001
Never	7,632 (59.20)	2,112 (65.61)	1,962 (60.97)	1,813 (56.34)	1,735 (53.92)	
Former	44,22 (34.30)	975 (30.29)	1.095 (34.03)	1,174 (36.48)	1,170 (36.36)	
Current	809 (6.27)	126 (3.91)	156 (4.85)	222 (6.90)	301 (9.35)	
Drinking status, *n* (%)						0.355
Never	356 (2.76)	88 (2.73)	80 (2.49)	91 (2.83)	97 (3.01)	
Former	305 (2.37)	73 (2.27)	80 (2.49)	63 (1.96)	89 (2.77)	
Current	12,230 (94.80)	3,057 (94.97)	3,057 (95.00)	3,063 (95.18)	3,031 (94.19)	
Physical activities, *n* (%)						<0.001
Low	1,983 (15.40)	405 (12.58)	486 (15.10)	510 (15.85)	581 (18.05)	
Moderate	4,436 (34.40)	1,139 (35.38)	1,056 (32.82)	1,123 (34.90)	1,107 (34.40)	
High	4,218 (32.70)	1,190 (36.97)	1,117 (34.71)	1,032 (32.07)	873 (27.13)	
BMI ≥ 30kg/m^2, *n* (%)	2.549 (19.80)	175 (5.44)	464 (14.42)	743 (23.09)	1,165 (36.20)	<0.001
Diabetes, *n* (%)	482 (3.74)	127 (3.95)	114 (3.54)	104 (3.23)	137 (4.26)	0.142

High blood pressure, *n* (%)	5.405 (41.90)	1,064 (33.05)	1,343 (41.73)	1,480 (45.99)	1,511 (46.95)	<0.001
Depression, *n* (%)	407 (3.16)	79 (2.45)	102 (3.17)	103 (3.20)	122 (3.79)	0.019
Cancer, *n* (%)	823 (6.38)	177 (5.50)	221 (6.87)	213 (6.62)	211 (6.56)	0.114
HDL-C, mean (SD), mmol/L	1.45 (0.37)	1.58 (0.39)	1.48 (0.37)	1.41 (0.35)	1.34 (0.34)	<0.001
LDL-C, mean (SD), mmol/L	3.55 (0.86)	3.20 (0.77)	3.51 (0.82)	3.68 (0.85)	3.80 (0.86)	<0.001
Cholesterol, mean (SD), mmol/L	5.67 (1.13)	5.28 (1.04)	5.62 (1.08)	5.82 (1.12)	5.96 (1.16)	<0.001
CRP, median (IQR), mg/L	1.16 (1.75)	0.42 (0.34)	0.91 (0.67)	1.57 (1.18)	3.59 (4.11)	<0.001
RC, median (IQR), mg/dL	24.56 (13.38)	18.95 (10.25)	23.43 (11.79)	27.07 (12.88)	29.66 (14.46)	<0.001
RCII, median (IQR)	2.91 (5.18)	0.81 (0.75)	2.19 (1.55)	4.42 (3.16)	11.02 (11.93)	<0.001

TDI, Townsend deprivation index; BMI, body mass index; SBP, systolic blood pressure; HDL-C, high-density lipoprotein cholesterol; LDL-C, low-density lipoprotein direct; Medication, medication for cholesterol, blood pressure, or diabetes; CRP, C-reactive protein; RC, remnant cholesterol; RCII, remnant cholesterol inflammatory index.

Table S4. Associations of RC, CRP, and RCII with the risk of frailty after being imputed.

Indicators	Model 1		Model 3	
	HR (95%*CI*)	*P* for trend	*HR* (95%*CI*)	*P* for trend
**RCII**		<0.001		<0.001
Q1	1 (reference)		1 (reference)	
Q2	1.41 (1.22-1.62)***		1.18 (1.02-1.36)*	
Q3	1.92 (1.68-2.20) ***		1.36 (1.18-1.56) ***	
Q4	2.92 (2.57-3.12) ***		1.67 (1.46-1.91) ***	
Per SD	1.16 (1.14-1.18) ***		1.09 (1.06-1.12) ***	
**RC**		<0.001		<0.001
Q1	1 (reference)		1 (reference)	
Q2	1.15 (1.02-1.30) *		1.06 (0.94-1.20)	
Q3	1.17 (1.04-1.32) *		1.01 (0.90-1.25)	
Q4	1.52 (1.35-1.70) ***		1.24 (1.10-1.40) ***	
Per SD	1.15 (1.11-1.19) ***		1.08 (1.04-1.12) ***	
**CRP**		<0.001		<0.001
Q1	1 (reference)		1 (reference)	
Q2	1.58 (1.37-1.82)***		1.32 (1.15-1.53) ***	
Q3	2.00 (1.75-2.30) ***		1.40 (1.22-1.61) ***	
Q4	3.14 (2.77-3.57) ***		1.78 (1.55-2.04) ***	
Per SD	1.16 (1.13-1.19) ***		1.08 (1.05-1.12) ***	

Model 1 unadjusted; Model 3 adjusted for age, sex, occupational status, income, ethnicity, education, TDI, drinking status, smoking status, BMI, physical activity, high blood pressure, diabetes, cancer, and depression. *** means P < 0.001; ** means P < 0.01; * means P < 0.05.

Table S5. Associations of CumRC, CumCRP, and CumRCII with the risk of frailty after being imputed.

Indicators	Model 1		Model 3	
	HR **(95%*CI*)**	***P* for trend**	***HR*** **(95%*CI*)**	***P* for trend**
**CumRCII**		<0.001		<0.001
Q1	1 (reference)		1 (reference)	
Q2	1.81 (1.30-2.51)***		1.48(1.06-2.06)*	
Q3	2.37 (1.73-3.25) ***		1.64 (1.19-2.27) **	
Q4	3.89 (2.89-5.24) ***		2.11 (1.54-2.88) ***	
Per SD	1.26 (1.21-1.31) ***		1.18 (1.13-1.24) ***	
**CumRC**		<0.001		<0.001
Q1	1 (reference)		1 (reference)	
Q2	1.27 (0.96-1.68)		1.19 (0.90-1.57)	
Q3	1.24 (0.93-1.63)		1.12 (0.84-1.48)	
Q4	1.97 (1.52-2.54) ***		1.65 (1.27-2.15)***	
Per SD	1.27 (1.18-1.37) ***		1.20 (1.11-1.30)***	
**CumCRP**		<0.001		<0.001
Q1	1 (reference)		1 (reference)	
Q2	1.58 (1.37-1.82)**		1.28 (0.91-1.79)	
Q3	2.35 (1.75-2.30) ***		1.56 (1.14-2.16) **	
Q4	3.94 (2.77-3.57) ***		2.05 (1.51-2.80) ***	
Per SD	1.27 (1.22-1.32) ***		1.18 (1.12-1.24) ***	

Model 1 unadjusted; Model 3 adjusted for age, sex, occupational status, income, ethnicity, education, TDI, drinking status, smoking status, BMI, physical activity, high blood pressure, diabetes, cancer, and depression. *** means P < 0.001; ** means P < 0.01; * means P < 0.05.

Table S6. Association of RC, CRP, and RCII with frailty risks when using the competing risk model.

**Indicators**	**Model 1**		**Model 3**	
	***HR* (95%*CI*)**	***P* for trend**	***HR* (95%*CI*)**	***P* for trend**
**RCII**		<0.001		<0.001
Q1	1 (reference)		1 (reference)	
Q2	1.40 (1.21-1.61)***		1.18 (1.03-1.37)*	
Q3	1.88 (1.65-2.15) ***		1.36 (1.18-1.56) ***	
Q4	2.80 (2.47-3.18) ***		1.64 (1.43-1.89) ***	
Per SD	1.15 (1.13-1.17) ***		1.08 (1.06-1.11) ***	
**RC**		<0.001		<0.001
Q1	1 (reference)		1 (reference)	
Q2	1.15 (1.02-1.30) *		1.07 (0.94-1.21)	
Q3	1.17 (1.04-1.32) *		1.03 (0.91-1.63)	
Q4	1.52 (1.35-1.70) ***		1.26 (1.12-1.42) ***	
Per SD	1.15 (1.11-1.19) ***		1.08 (1.04-1.13) ***	
**CRP**		<0.001		<0.001
Q1	1 (reference)		1 (reference)	
Q2	1.57 (1.36-1.80)***		1.33 (1.15-1.53) ***	
Q3	1.96 (1.71-2.24) ***		1.40 (1.21-1.61) ***	
Q4	2.99 (2.63-3.40) ***		1.73 (1.51-1.99) ***	
Per SD	1.15(1.13-1.17) ***		1.07 (1.04-1.10) ***	

Model 1 unadjusted; Model 3 adjusted for age, sex, occupational status, income, ethnicity, education, TDI, drinking status, smoking status, BMI, physical activity, high blood pressure, diabetes, cancer, and depression. *** means P < 0.001; * means P < 0.05.

Table S7. Association of CumRC, CumCRP, and CumRCII with frailty risks when using the competing risk model

Indicators	Model 1		Model 3	
	***HR* (95%*CI*)**	***P* for trend**	***HR* (95%*CI*)**	***P* for trend**
**CumRCII**		<0.001		<0.001
Q1	1 (reference)		1 (reference)	
Q2	1.80 (1.30-2.51)***		1.48(1.06-2.07)*	
Q3	2.36 (1.72-3.24) ***		1.65 (1.19-2.29) **	
Q4	3.87 (2.87-5.21) ***		2.11 (1.54-2.90) ***	
Per SD	1.26 (1.20-1.31) ***		1.18 (1.13-1.24) ***	
**CumRC**		<0.001		<0.001
Q1	1 (reference)		1 (reference)	
Q2	1.27 (0.96-1.68)		1.27 (0.96-1.68)	
Q3	1.24 (0.93-1.63)		1.24 (0.94-1.64)	
Q4	1.97 (1.52-2.54) ***		1.97 (1.53-2.54)***	
Per SD	1.27 (1.19-1.36) ***		1.21 (1.12-1.30)***	
**CumCRP**		<0.001		<0.001
Q1	1 (reference)		1 (reference)	
Q2	1.58 (1.13-2.21)***		1.27 (0.90-1.77)	
Q3	2.35 (1.71-3.21) ***		1.58 (1.14-2.18) ***	
Q4	3.91 (2.92-5.25) ***		2.04 (1.50-2.78) ***	
Per SD	1.27 (1.22-1.31) ***		1.18 (1.13-1.24) ***	

Model 1 unadjusted; Model 3 adjusted for age, sex, occupational status, income, ethnicity, education, TDI, drinking status, smoking status, BMI, physical activity, high blood pressure, diabetes, cancer, and depression. *** means P < 0.001; ** means P < 0.01; * means P < 0.05.

Table S8. Association of RCII and CumRCII with frailty risk based on restricted cubic splines categories

Group	n	Model 1	Model 2	Model 3
		HR (95%CI)	HR (95%CI)	HR (95%CI)
RCII <3.32	204,060	1 (reference)	1 (reference)	1 (reference)
RCII ≥3.32	198,790	1.03 (1.01-1.05) **	1.04 (1.01-1.07) **	1.05 (1.02-1.08)**
CumRCII <13.60	6,478	1 (reference)	1 (reference)	1 (reference)
CumRCII ≥13.60	6,417	2.24 (1.86-1.71) ***	1.43 (1.10-1.86) **	1.61 (1.22-2.12)***

Model 1 unadjusted; Model 3 adjusted for age, sex, occupational status, income, ethnicity, education, TDI, drinking status, smoking status, BMI, physical activity, high blood pressure, diabetes, cancer, and depression. *** means P < 0.001; ** means P < 0.01.
